# An XIST-related small RNA regulates KRAS G-quadruplex formation beyond X-inactivation

**DOI:** 10.18632/oncotarget.13433

**Published:** 2016-11-17

**Authors:** Yuli C. Chang, Chien-Chih Chiu, Chung-Yee Yuo, Wen-Ling Chan, Ya-Sian Chang, Wen-Hsin Chang, Shou-Mei Wu, Han-Lin Chou, Ta-Chih Liu, Chi-Yu Lu, Wen-Kuang Yang, Jan-Gowth Chang

**Affiliations:** ^1^ Graduate Institutes of Medicine, College of Medicine, Kaohsiung Medical University, Kaohsiung, Taiwan; ^2^ Division of Cytogenetics, Department of Laboratory Medicine, Kaohsiung Medical University Hospital, Kaohsiung, Taiwan; ^3^ Department of Biotechnology, Kaohsiung Medical University, Kaohsiung, Taiwan; ^4^ Epigenome Research Center, China Medical University and Hospital, Taichung, Taiwan; ^5^ School of Pharmacy, College of Pharmacy, Kaohsiung Medical University, Kaohsiung, Taiwan; ^6^ Department of Biomedical Science and Environmental Biology, Kaohsiung Medical University, Kaohsiung, Taiwan; ^7^ Division of Hematology/Oncology, Department of Internal Medicine, Kaohsiung Medical University Hospital, Kaohsiung, Taiwan; ^8^ Department of Bioinformatics and Medical Engineering, Asia University; ^9^ Department of Biochemistry, College of Medicine, Kaohsiung Medical University, Taiwan; ^10^ Cell/Gene Therapy Research Laboratory, Department of Medical Research, China Medical University Hospital, Taichung, Taiwan; ^11^ Department of Laboratory Medicine, China Medical University Hospital, Taichung, Taiwan; ^12^ School of Medicine, China Medical University, Taichung, Taiwan

**Keywords:** XPi2, non-coding RNA, *XIST*, G-quadruplexes

## Abstract

X-inactive-specific transcript (*XIST*), a long non-coding RNA, is essential for the initiation of X-chromosome inactivation. However, little is known about other roles of *XIST* in the physiological process in eukaryotic cells. In this study, the bioinformatics approaches revealed *XIST* could be processed into a small non-coding RNA XPi2. The XPi2 RNA was confirmed by a northern blot assay; its expression was gender-independent, suggesting the role of XPi2 was beyond X-chromosome inactivation. The pull-down assay combined with LC-MS-MS identified two XPi2-associated proteins, nucleolin and hnRNP A1, connected to the formation of G-quadruplex. Moreover, the microarray data showed the knockdown of XPi2 down-regulated the *KRAS* pathway. Consistently, we tested the expression of ten genes, including *KRAS*, which was correlated with a G-quadruplex formation and found the knockdown of XPi2 caused a dramatic decrease in the transcription level of *KRAS* among the ten genes. The results of CD/NMR assay also supported the interaction of XPi2 and the polypurine-polypyrimidine element of *KRAS*. Accordingly, XPi2 may stimulate the *KRAS* expression by attenuating G-quadruplex formation. Our present work sheds light on the novel role of small RNA XPi2 in modulating the G-quadruplex formation which may play some essential roles in the *KRAS*- associated carcinogenesis.

## INTRODUCTION

X chromosome inactivation (XCI) is a process associated with gene dosage compensation between XX and XY individuals [1, 2]. This epigenetic process inactivates one of the two X chromosomes in females [3]. There is only one copy of each X-linked gene active in female cells (XX), and male cells carry only one X chromosome (XY) [4]. There have been some new mechanistic advances addressed in mouse and human XCI biology recently, for example, the role of XCI in cancer initiation or the ability to mark XCI in individual cells [5].

The *XIST* gene is localized within the region on the X chromosome known to contain the X-inactivation center (XIC) [6]. The *XIST* gene is expressed exclusively from the XIC of the inactive X chromosome to equalize gene expression in males and females [7]. Apart from monoallelic expression in the process of XCI, the *XIST* gene is influenced by a master control region and is associated with multiple long noncoding RNAs (lncRNA) [4]. *XIST* contains at least eight exons with a total length of 17 kb [6], and the noncoding RNA *XIST* is a chief regulator acting as a major effector central to the XCI phenomenon [3, 4, 8].

Noncoding RNAs (NcRNAs) may contain different varieties [9] and most ncRNAs operate as RNA-protein complexes, including ribosomes, telomerase, small nucleolar RNAs (snoRNAs), small nuclear RNAs (snRNAs), small interfering RNA (siRNA), microRNAs, PIWI-interacting small RNAs (piRNAs) and long ncRNAs [9, 10].

Ogawa et al. explored a role for RNAi in XCI to identify small RNAs within *Xist/Tsix* [11]. Brown et al. reported that the non-coding RNA *XIST* could function as a cis-acting silencer in different cell lines [12]. Thus, small ncRNAs could be generated from *Xist* [3], and those small RNA may participate in the intersection of the RNA interference and X-inactivation pathways [3, 11, 13]. Furthermore, *Xist* A-repeat small RNA can cause the silencing of genes, such as *Lamp2*, *MeCP2*, *G6pdx*, and *Chic1* [14]. A recent study has shown that the knockdown of long non-coding RNA *XIST* suppressed tumor functions by upregulating miR-152 [15]. It has been suggested that dysfunctional expression of *XIST* may have a pathological role in cancer, possibly associated with changes in gene expression from alterations in the stability of heterochromatin [16].

An *XIST*- correlated small RNA named XPi2 was present in the piRNA bank. In this study, we identified two XPi2-associated proteins, nucleolin [17, 18], and hnRNP A1 [19], by the pull-down assay combined with LC-MS, and both proteins were connected to the formation of G-quadruplex [20]. Furthermore, PIWI-associated RNAs were also found to be aberrantly expressed in human somatic tumors, implying that the PIWI pathway has a more profound function outside germline cells than was originally thought [21]. For example, PIWI*-*interacting *RNA 021285* is involved in breast tumorigenesis possibly by remodeling the cancer epigenome [22].

G-quadruplex DNA (also known as G4 DNA) is composed of four independent DNA strands that interact closely with other structures to form specific tetramolecular quadruplexes. G-quadruplex DNAs have numerous functions, such as conserving telomere length and integrity, regulating transcription, and processing biological conditions that influence malignant cell behavior [23–26].

The *KRAS* gene was sieved out as a potential XPi2-regulated gene by our cDNA microarray results. The *KRAS* amplification, as well as its mutations, can induce cells to divide in response to growth factors, which results in over-activation of cell proliferation [27]. It has been proposed that G-quadruplex formation within the *KRAS* gene may affect its transcription [28].

Our study showed that this small RNA was involved in the change of the G-quadruplex structure to alter gene expression. We demonstrated the existence of a small RNA XPi2 from the non-coding *XIST* gene, which plays an essential role in altering *KRAS* expression. Furthermore, the clinical pathology of XPi2 in cellular tumorigenesis was also discussed.

## RESULTS

### Identification of XPi2

**Figure 1:**
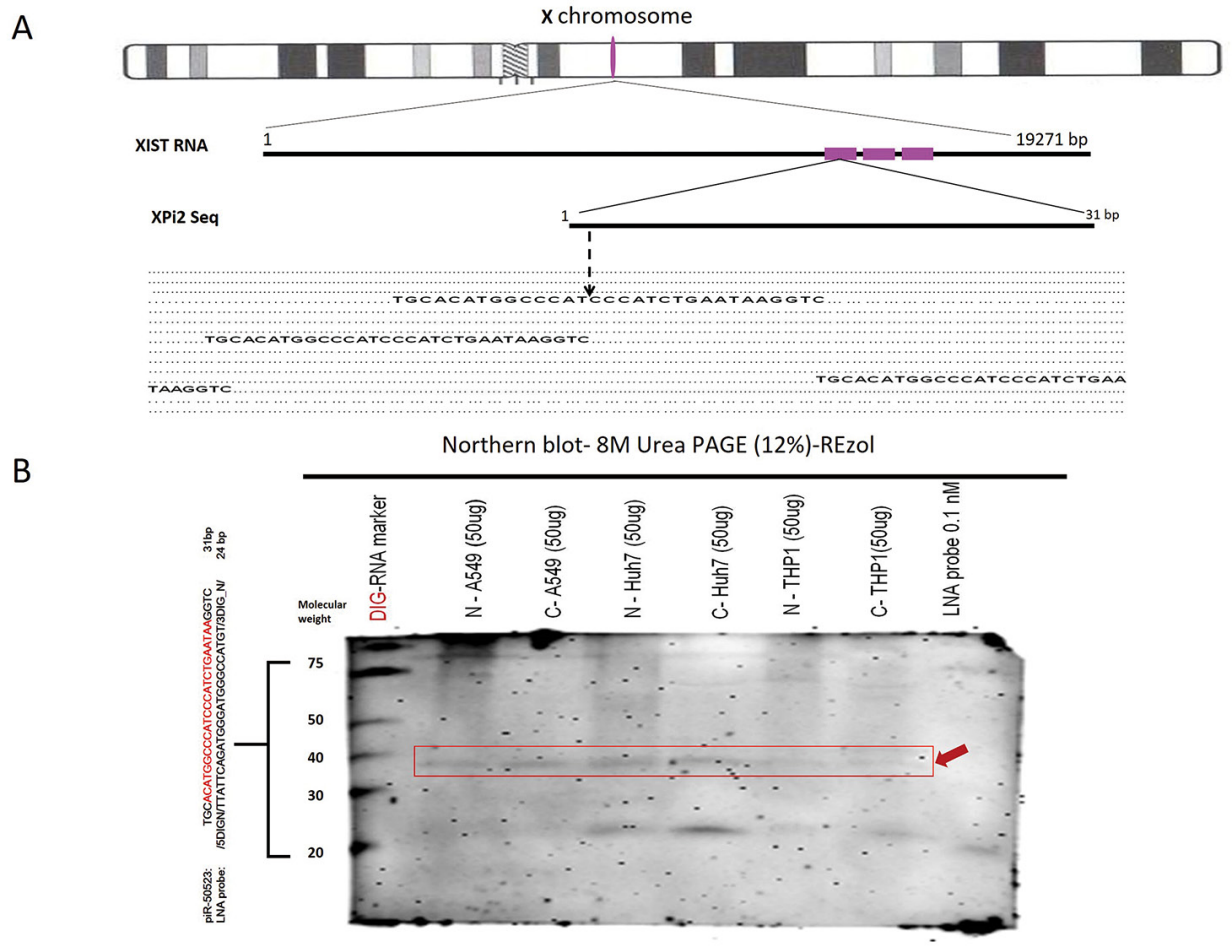
Existence and Localization of XPi2 **A**. The location of XPi2 on chromosome X. The alignment of XPi2 was performed by comparing the sequences of XPi2 and *XIST*. These three repeat XPi2 sequences (red) were located in the *XIST* gene. **B**. Northern blots showed the XPi2 probe (piR50523 DIG LNA-probe: /5DigN/TTATTCAGATGGGATGGGCCATGT/3Dig_N/) shifted in different cell line conditions by different fractions between cytoplasm and nucleus (N= nucleus, C= cytoplasm). The labels have also been aligned as follows accordingly: the DIG-RNA marker, N –A549, C-A549, N-Huh7, C-Huh7, N-THP1, C-THP1, and LNA probe 0.1nM only, respectively. (Loading control: LNA probe 0.1nM only; Red arrowhead: XPi2; Nonspecific band on 21 molecular weight)

In this study, we used bioinformatics methods to obtain XPi2 from the BioMart database and integrated the information in the Ensembl Genome Browser. According to our previous study, we aligned the sequences of fsRNAs and found this pi-like RNA molecule was located in the X-inactive specific transcript (*XIST)* region [29]. A small 31nt RNA was found, and it was determined that three repeat sequences of XPi2 were present on the repeat A portion of the *XIST* RNA (depicted red) (Figure 1A). Furthermore, the northern blot method is a convincing method for small RNA validation and an enhanced detection way to improve the existence of small RNA molecules [30, 31]. In this study, the existence of XPi2 was detected and confirmed by northern blots (Figure 1B). We also propose that the presence of XPi2 is important in human evolution as supported by the data that XPi2 is highly conserved among mammalian species (Supplementary Figure S1-1A, S1-1B).

### The significance of XPi2 biogenesis

**Figure 2:**
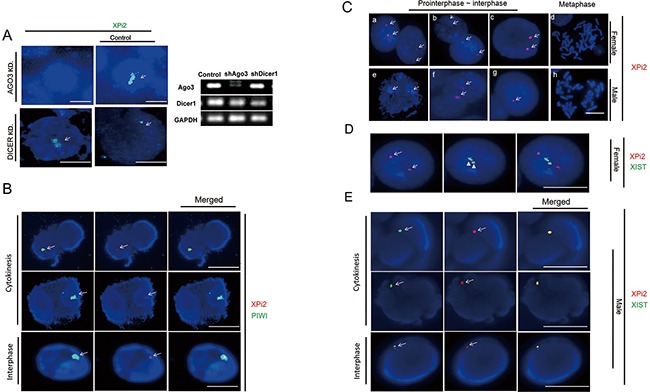
The relationships between XPi2, PIWIL1, and *XIST* were explored, respectively **A**. XPi2 was produced through the PIWI pathway about Argonaut. After Argonaut had been knocked down, the expression of XPi2 was markedly decreased, as shown by FISH using the XPi2 probe (green), in the Huh-7 cell line. After *DICER* had been knocked down, there was no visible change in the XPi2 expression. **B**. The Huh-7 cell line (JCRB0403) was subjected to FISH analysis using the XPi2 probe (red) and immunofluorescence staining using the anti-PIWIL1 antibody (green), and 100 cells were Colcemid stimulated and analyzed. 40% of the total cells (the result only showed colocalization in both interphase and cytokinesis) showed the same localization between PIWI and XPi2. **C**. Normal female cells (46, XX) and male cells (46, XY) in different stages of the cell cycle were subjected to FISH analysis using the XPi2 probe (red). a, b, c, e, f, and g represented positive signals in cells during interphase, whereas d and h showed negative signals in cells during metaphase. **D**. (1500X Magnification) Lymphoid cells were subjected to locked nucleic acid-FISH with enzyme-labeled fluorescence, and 100 cells were counted. The XPi2 probe was labeled with CY5 (red), and the *XIST* probe was labeled with FITC (green). **E**. The Huh-7 cell line was subjected to both XPi2 and *XIST* RNA-FISH probes with labeled fluorescence and 100 cells were counted. The XPi2 RNA probe was labeled with CY5 (red), and the *XIST* RNA probe was labeled with FAM (green) showing different fluorescence intensities. Different cell stages showed different degrees of colocalization which demonstrated different processing stages of XPi2 RNA in the nucleus from those of the result in Figure (D). (100 cells showed 30 % colocalization in cytokinesis, 25% colocalization in interphase, and 0% in metaphase).

In order to determine whether XPi2 was produced through the PIWI pathway, we used shRNA to knock down the enzymes involved in the miRNA and PIWI pathways. Argonaut 3 (Ago3) is a key element of the PIWI pathway, which has been reported as an RNA silencing pathway of Drosophila [32–35]. We measured the expression of XPi2 by the fluorescence *in situ* hybridization (FISH) assay with antisense LNA (locked nucleic acid) and RNA probes. The results showed that the expression of XPi2 markedly decreased after the knockdown of *AGO3* and detected by an AGO3 antibody described in the experiment section (Figure 2A). On the contrary, the expression of XPi2 remained unchanged after the knockdown of *DICER* (Figure 2A). Also, we used FISH and immunofluorescence staining to show the co-localization of XPi2 with PIWI, an essential protein of the PIWI pathway (Figure 2B). Based on the above results, we suggest that XPi2 was produced through the PIWI pathway.

We also used the FISH assay to determine the cellular localization of XPi2 during the different stages of the cell cycle. We found that XPi2 was localized in the nucleus of most cells during interphase and cytokinesis but not metaphase (Figure 2C). These results suggest that XPi2 may activate or suppress its targets in the nuclei. Also, since both female and male cell lines showed the similar results, the data also indicated that XPi2 might not be involved in the *XIST* beyond X chromosome inactivation (XCI).

To further explore the relationship between XPi2 and *XIST*, we measured the expression levels of XPi2 and *XIST* in female and male cell lines by quantitative RT-PCR. The results showed that the expression level of XPi2 did not directly correlate with that of *XIST* and was unrelated to sexuality (Supplementary Table S1 and Figure S1-3). Moreover, we used locked nucleic acid-FISH with enzyme-labeled fluorescence (LNA-ELF-FISH) to analyze the localization of XPi2 and *XIST* in female lymphoid cells. We found that there was no consistency in the intracellular locations of XPi2 and *XIST* in female lymphoid cells (Figure 2D), but there was colocalization in the JCRB0403 male cell line (Figure 2E). The presence of the FISH data of the single molecule XPi2 combined with *XIST*, together with the intracellular localization data in Figures 2D and 2E suggested that XPi2 could exist in the nucleus where it might bind or unbind *XIST* during different cell phases and at different DNA or RNA levels.

**Figure 3:**
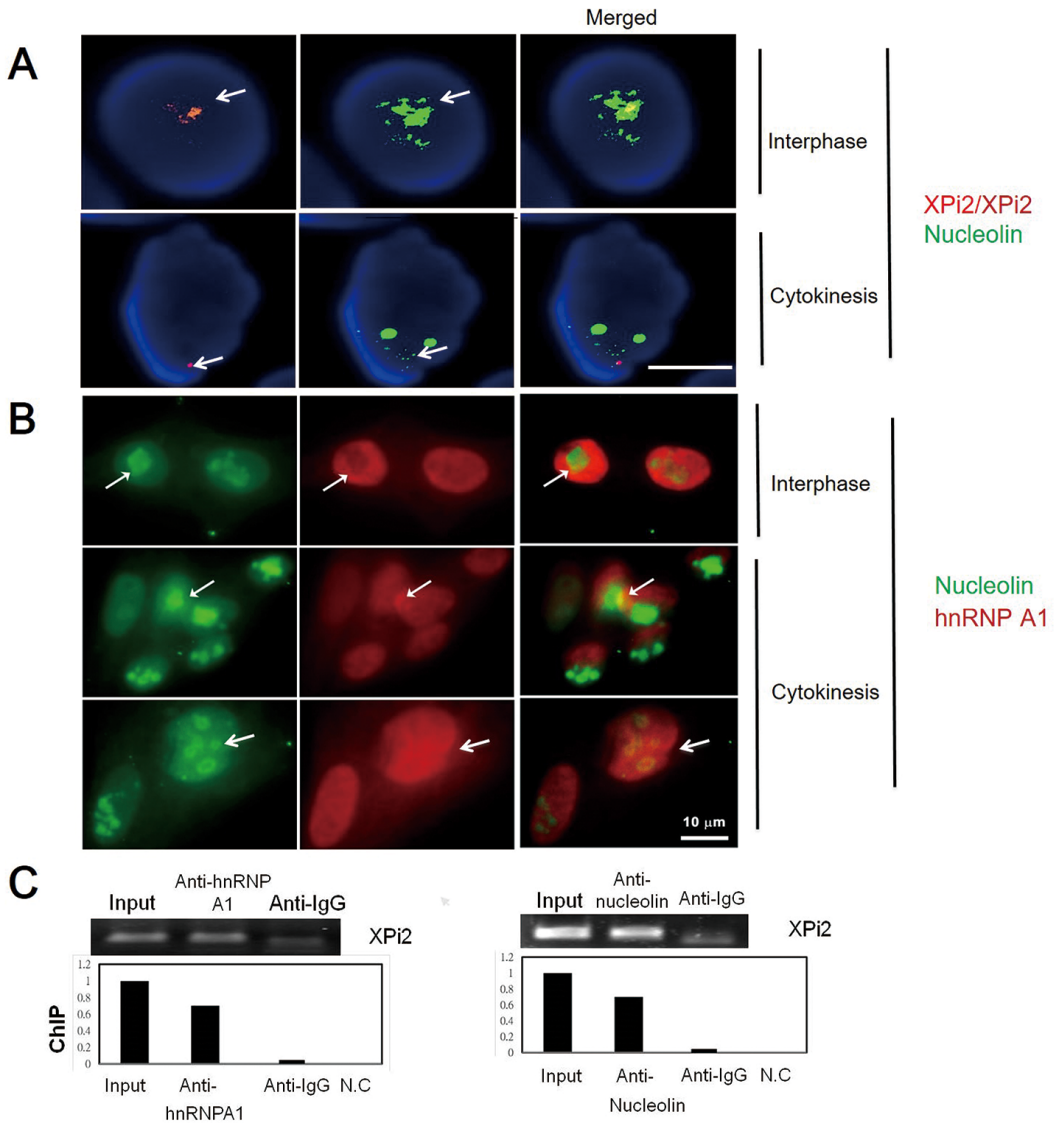
Interactions between nucleolin, hnRNP A1, and XPi2 were confirmed by co-localization and ChIP analyses **A**. The Huh-7 cell line was subjected to FISH analysis using the XPi2 probe (red) and immunofluorescence staining using the anti-nucleolin antibody (green). The result showed that XPi2 co-localized with nucleolin (100 cells were analyzed, and 40% cells showed co-localization in different cell stages, interphase, and cytokinesis, respectively). **B**. The Huh-7 cell line was subjected to immunofluorescence staining using the anti-nucleolin (green) and anti-hnRNP A1 (red) antibodies. The result showed the co-localization of nucleolin and hnRNP A1 in interphase and cytokinesis. **C**. Chromatin immunoprecipitation (ChIP) was performed using anti-hnRNP A1 (left panel) or anti-nucleolin (right panel). The immunoprecipitates were subjected to qRT-PCR to analyze XPi2 RNA. The positive input, anti-IgG, and negative control (NC) were also included in the analysis as controls. The results showed that XPi2 was directly bound to nucleolin and hnRNP A1. (The result of DNA sequencing confirmed XPi2 after ChIP, data not shown)

Consequently, we suggest that XPi2 may play a functional role beyond X-chromosome inactivation.

### Identification of nucleolin and hnRNP A1 as XPi2-associated proteins

**Figure 4:**
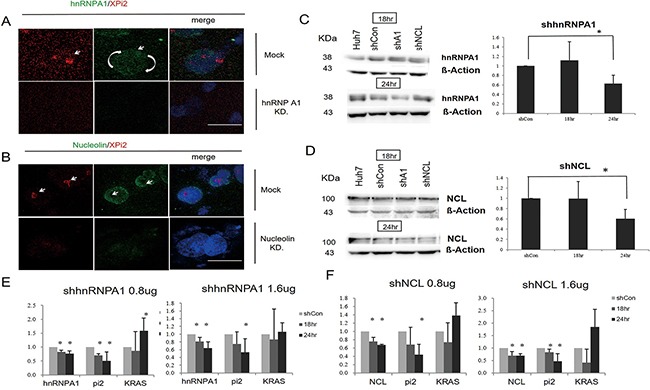
Knockdown of either hnRNP A1 or nucleolin disrupted the XPi2-nucleolin-hnRNP A1 complex **A**. The Huh-7 cell line was transfected with the shRNA vector targeting *hnRNP A1* (KD) or PLKO.1-shLuc control vector (mock). Then, the transfected cells were subjected to FISH analysis using the XPi2 probe (red) and immunofluorescence staining using the anti-hnRNP A1 antibody (green). **B**. The Huh-7 cell line was transfected with the shRNA vector targeting *nucleolin* (KD) or PLKO.1-shLuc control vector (mock). Then, the transfected cells were subjected to FISH analysis using the XPi2 probe (red) and immunofluorescence staining using the anti-nucleolin antibody (green). **C**. Western blot analyses in Huh7, the shscramble control, sh*A1*(sh*hnRNP A1)*, and sh*NCL* (*nucleolin*): Four different conditions were treated with the anti-hnRNP A1 antibody. The cells were transfected in 18 and 24 hrs, respectively. B-Actin was an internal control. Quantification analysis: Data presented as mean ± S.D. in triplicate. Asterisks indicated statistically significant differences compared with those of the control (*P < 0.05 for the control versus each treatment, respectively). **D**. Western blot analyses in Huh7, the shscramble control, sh*hnRNPA1*, and sh*NCL*: Four different conditions were treated with the anti-nucleolin antibody. The cells were transfected in 18 and 24 hrs, respectively. B-Actin was an internal control. Quantification analysis: data presented as mean ± S.D. in triplicate. Asterisks indicated statistically significant differences compared with those of the control (*P < 0.05 for the control versus each treatment, respectively). **E**. The results of the quantification analysis in *hnRNP A1*, XPi2 (Pi2), and *KRAS* after sh*hnRNPA1* was treated. (*P < 0.05, * *P < 0.001 for the control versus each treatment with different doses of sh*hnRNP A1* at 18 and 24 hrs) **F**. The results of the quantification analysis in NCL (*nucleolin*), XPi2 (Pi2), and *KRAS* after sh*NCL* was treated. (*P < 0.05, * *P < 0.001 for the control versus each treatment with different doses of sh*NCL* at 18 and 24 hrs)

To investigate the function of XPi2, we used XPi2 RNA as a probe to isolate its binding proteins by pull-down assays and mass spectrometry (LC-MS/MS). Two XPi2-associated proteins, nucleolin and hnRNP A1, were identified (Supplementary Table S2). To further confirm the correlation among nucleolin, hnRNP A1, and XPi2, we performed FISH and immunofluorescence staining to determine their co-localization. The results demonstrated that XPi2 co-localized with nucleolin, and that hnRNP A1 and nucleolin co-localized with each other (Figure 3A and 3B). Nucleolin has been reported to be involved in the biogenesis of microRNA, and another protein, hnRNP A1, is now critical for the biological process of XPi2 in our study. We found that cellular localization was critical for the proper functioning of nucleolin and hnRNP A1 in this pathway, and that nucleolin and hnRNP A1 directly interacted with XPi2 in the nucleus (3A, B). To test our hypothesis and finding, we carried out the knockdown studies in the next step. Quantitative chromatin immunoprecipitation (ChIP) was performed to investigate the *in vivo* binding of XPi2 with hnRNP A1 and nucleolin. The ChIP results confirmed that XPi2 was directly bound to each protein (Figure 3C). Also, we also found that the XPi2-nucleolin-hnRNP A1 complex disassembled, and XPi2 was degraded after the knockdown of either *hnRNP A1* or *nucleolin* (Figure 4A and 4B). There was a decrease in the intracellular levels of XPi2 after *hnRNP A1* was knocked down (Figure 4A) compared with the mock transfected cells. The same decrease in the intracellular levels of XPi2 after *nucleolin* was knocked down (Figure 4B) was observed compared with the mock transfected cells. When the cells were transfected, western blots were performed for hnRNP A1 and nucleolin. The knockdown of *hnRNP A1* (sh*hnRNA A1*) led it to significantly decrease at 24h, and the same situation happened when *nucleolin* was knocked down. It was expected that when *hnRNP A1* and *nucleolin* were knocked down, they would inhibit their target, XPi2 (Figures 4E, 4F). The results showed that the knocked-down proteins in transfected cells showed a decrease in the expression of XPi2 (Figures 4E, 4F). Therefore, our results indicated that XPi2 was associated with nucleolin and hnRNP A1, which might be important for the function of XPi2 (Figures 4C, 4D, 4E, and 4F).

### Knockdown of XPi2 affected the genes in the RAS pathway

Subsequently, we performed a genome-wide gene expression microarray analysis of XPi2-knockdown Huh-7 cells. As a result, 77 genes were up-regulated, and 69 genes were down-regulated with a greater than a 2-fold change in XPi2-knockdown cells compared with control cells (Supplementary Figure S1-1). Gene ontology analysis of the down-regulated genes revealed two significant pathways, telomere maintenance and Sprouty regulation of tyrosine kinase signals (Supplementary Figure S1-2). We excluded the relation between telomere and XPi2 from the FISH data (data not shown). The Sprouty (S*PRY*) protein inhibits the Ras/mitogen-activated protein (MAP) kinase pathway induced by various activated receptor tyrosine kinases [26].

### XPi2 altered *KRAS* expression and correlated with the *KRAS* promoter

**Figure 5:**
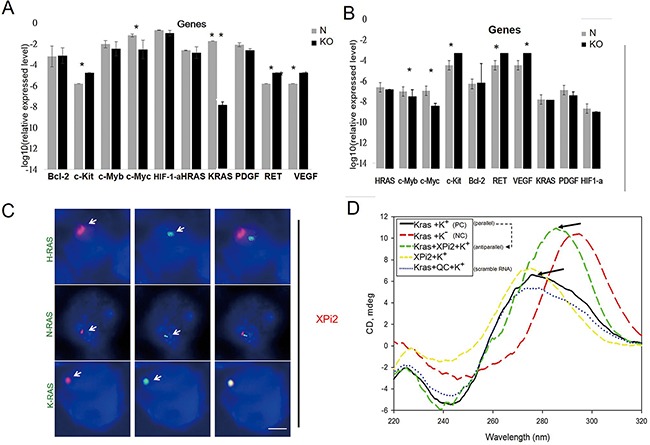
XPi2 regulates *KRAS* expression and interacts with the *KRAS* promoter **A**. The huh-7 cell line was treated with the shXPi2 (KO) or scrambled control oligonucleotide (N). Then, the expressional changes of ten quadruplex-associated genes, *KRAS*, *C-MYC*, *C-MYB*, *HRAS, BCL-2, RET, PDGF, VEGF, HIF-1-α*, and *C-KIT* were determined by qRT-PCR after the knockdown of XPi2. Significant decreases in the levels of *KRAS* were detected. The result of the quantification analysis in each gene after shXPi2 was treated. (*P-value < 0.05, * * P-value < 0.001 for the control versus each gene after the knockdown treatment in the Huh7 cell line). **B**. The same condition as (A) except using the MCF cell line: The result of the quantification analysis in each gene after shXPi2 was treated. (*P < 0.05, * *P < 0.001 for the control versus each gene after the knockdown treatment in the MCF cell line). **C**. The Huh-7 cell line was subjected to FISH analysis using the XPi2 RNA-FISH probe (red) and the DNA- FISH probes (green) for the promoter sequences of *KRAS*, *HRAS*, and *NRAS*. The result showed the co-localization of XPi2 and the *KRAS* promoter, but not the *HRAS* and N*RAS* promoters. **D**. XPi2 RNA, *KRAS* DNA, and XPi2-*KRAS* complex were analyzed by CD analysis. The samples contained 50 μM XPi2 RNA and *KRAS* DNA in a solution of 50 mM Tris-HCl, pH 7.2 and 100 mM KCl, respectively. The spectra were recorded in a 5-cm quartz cuvette. They were accumulated from left top 260nm to the right bottom 280nm in K+ condition (parallel - black line), and then added the XPi2 RNA shift to further right from 280nm to 300nm (antiparallel - green line) when XPi2 RNA was added. (QC=scramble RNA; NC=without K condition) Guanines in the parallel-stranded G-quadruplex have exhibited a positive peak at 260 nm and a small negative peak at 240 nm in the CD spectra. When switched to the antiparallel-stranded, there was a positive peak at 280nm-320nm, in which the formation of a s-quadruplex structure is in K+ and at room temperature. When XPi2 is present, the peak is the same as that without potassium [54].

Recent studies have shown that both nucleolin and hnRNP A1 play a major role in G-quadruplex formation. The XPi2-nucleolin-hnRNP A1 complex may compose part of the G-quadruplex; therefore, altering this complex may influence gene expression. Thus, we selected ten quadruplex-associated genes, including *KRAS*, *C-MYC, C-MYB*, *HRAS, BCL-2, RET, PDGF, VEGF, HIF-1-α*, and *C-KIT*, and analyzed their expression by qRT-PCR after the knockdown of XPi2 in different cell lines (Figures 5A and 5B). The results showed that the expression level of *KRAS* changed significantly after the knockdown of XPi2 in Huh-7 cells.

To further confirm the role of XPi2 in regulating *KRAS* expression, we performed FISH analysis to determine whether XPi2 was localized to the *KRAS* promoter region. For comparison, the *HRAS* and *NRAS* promoters were also included in the study. We found that XPi2 specifically bound to the promoter of *KRAS* but not with the promoters of *HRAS* and *NRAS* (Figure 5C). Also, no change of XPi2 with the other quadruplex-associated genes was observed after *hnRNP A1* was knocked down (Supplementary Figure S2-1). Thus, our results indicate that XPi2, probably in the XPi2-nucleolin-hnRNP A1 complex, bound to the *KRAS* promoter and altered *KRAS* expression.

### Analysis of the interaction between XPi2 and *KRAS* G-quadruplex by FISH, circular dichroism (CD), and nuclear magnetic resonance (NMR)

Our results showed that XPi2 bound with nucleolin and hnRNP A1, which may be parts of the G-quadruplex, and XPi2 also bound with the *KRAS* promoter, which can form a G-quadruplex. We then tested whether XPi2 was involved in the formation of the *KRAS* G-quadruplex. We used a *KRAS*-G-quadruplex-specific probe (modified from Xu et al. 2010) to detect the G-quadruplex structure by FISH analysis after changing the XPi2-nucleolin-hnRNP A1 complex [36]. The results showed that the *KRAS* G-quadruplex probe reacted with XPi2, nucleolin, and hnRNP A1, but not hnRNP K. Also, XPi2 disrupted the interaction of the *KRAS* G-quadruplex with hnRNP A1 and nucleolin as well as the subsequent formation of a change-density *KRAS* quadruplex (Supplementary Figure S2-2). The result of the XPi2-*KRAS-*G-quadruplex complex with or without the knockdown of *hnRNP A1* or *nucleolin* is in accordance with our suggestion that XPi2 may work with nucleolin and hnRNP A1 as a key to change quadruplex structures and activation.

To determine the molecularity of the G-quadruplex, we performed CD and NMR analyses. CD is a well-known technique for characterizing the formation of G-quadruplex. When the small XPi2 RNA folds into the compact G-quadruplex structures, the CD spectra will show a different chemical shift. The result of the CD analysis indicated that the XPi2 RNA likely destabilized the formation of parallel to antiparallel *KRAS*-G-quadruplex structures (Figure 5D). The NMR analysis measures the exchange rate of the complex formation. The *KRAS*-G structures and the XPi2 RNA are in equilibrium between their free and bound states, and this equilibrium depends on the dissociation constant of the complex. The NMR result supported the interaction between XPi2 and the polypurine-polypyrimidine element of *KRAS* (Supplementary Figure S3).

### The role of XPi2 in cell proliferation and migration

To explore the biological functions of XPi2 RNA, we performed cell proliferation and migration assays on XPi2-knockdown (synthesized shRNA) in Huh-7 cells. We found that after the knockdown of XPi2, cell proliferation was decreased, and the cell migration activity was slightly up-regulated (Supplementary Figure S4). Also, ectopic expression of XPi2 could counteract the inhibitory effects of the XPi2 knockdown on Huh-7 cell proliferation (Supplementary Figure S4).

### XPi2 alters *KRAS* expression in colon cancer patients

**Figure 6:**
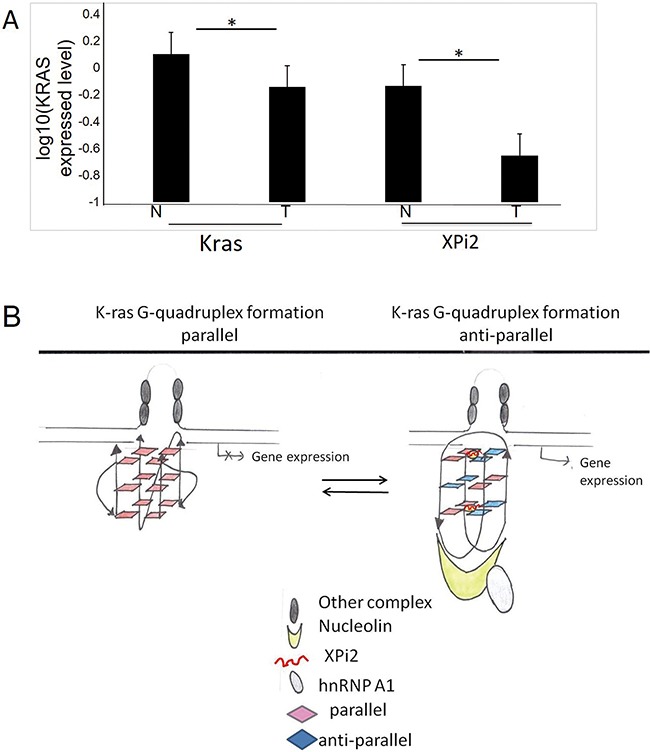
The comparison between XPi2 and *KRAS* expression in normal and tumor tissues **A**. Quantitative RT-PCR was performed to measure the expression levels of XPi2 RNA and *KRAS* mRNA in the normal and tumor parts of the 31 decoded samples of colon cancer patients. We found significantly reduced expression of both XPi2 and *KRAS* in colon carcinoma compared with that of the matched non-tumor tissues. T-test statistical analysis was performed. The P-value <0.05 (0.05*) indicated a significant correlation between *KRAS* and XPi2 expression levels in the normal and tumor parts of colon cancer. **B**. The proposed model of the XPi2 mechanism: The binding of the XPi2–mediated proteins, hnRNP A1 and nucleolin, with the structure of G-quadruplex changed the linearity of G-quadruplex inside cells.

We next explored the relationship between XPi2 and the *KRAS* gene in colon cancer patients. The *KRAS* gene had been shown to be expressed and mutated in colon cancer [37]. We analyzed the expression of *KRAS* and XPi2 in 31-colon cancer cases to determine their roles in the molecular pathogenesis of human colon cancer. A total of 31-colon cancer tissues and their paired nearby non-tumor tissues were collected from 31 patients, and all the patients had not received any preoperative chemotherapy or radiotherapy when their tissues were collected. The results showed that the expression of both *KRAS* and XPi2 was significantly reduced in colon carcinoma compared with that in the matched non-tumor tissues (Figure 6A). Statistical analysis indicated a significant correlation between *KRAS*, XPi2, and colon cancer in the normal and tumor tissues. Nonetheless, the mechanism underlying a role of XPi2 in colon cancer requires further research.

Our results showed that nucleolin, hnRNP A1, and XPi2 are pervasive in the G-quadruplex complex of specific oncogene and play important function roles in changing structure types of G-quadruplex. We, therefore, proposed a model of XPi2 that could be used to predict the assembly mechanism of the G-quadruplex structure (Figure 6B). The stability change of the G-quadruplex structure was determined by the existence of XPi2 RNA. We proposed that the combination of XPi2 RNA and G-quadruplex could transform parallel quadruplex structures into anti-parallel ones that might guide the quadruplex-targeted oncogene with different expression levels, as proved by clinical cases.

## DISCUSSION

Studies on transcriptional inactivation of the X chromosome in female mammals’ cells revealed important advances in our understanding of RNA biology in epigenetic control. Many of the mechanisms of X chromosome inactivation have been extensively studied and proved [5]. Brown *et al*. has reported that a gene (*Xist*) from the region of the human X inactivation center is expressed exclusively from the inactive X chromosome [2]. *Xist* RNA can spread along the length of the entire X chromosome without silencing active areas of the chromosome [5]. Those active regions may escape from X chromosome inactivation by looping out and remaining active while still permitting the spatial spread of *Xist*. In this study, we found that a small RNA generated from *Xist* may participate in changing G-quadruplex structures to control *KRAS* gene expression (Figures 4E and 4F, 5A, and 6A).

Small ncRNA could be produced from the *Xist* locus and generated from *Xist* [3, 11, 13]. The *Xist* RNA contains a region of conservation, the A-repeat-region, whose functions are still uncertain. It was shown that silencing of genes could be affected by the A repeat region of the *XIST* [38]. In contrast, some studies found that the RNA interference (RNAi) pathway was not essential for the initiation of X inactivation [39]. PIWI-interacting RNAs (piRNAs), one of the ncRNA categories [40], and Piwi proteins correlate with various essential biological functions [41, 42]. Also, PIWI-interacting RNAs (piRNAs) guide PIWI proteins to suppress transposons in the cytoplasm and nucleus of animal germ cells [43]. However, the mechanisms between piRNAs and proteins remain unclear. We found the existence of a small RNA, XPi2, which is located in *XIST*, via DNA, RNA, and LNA FISHs to detect the location of both XPi2 and *XIST* (Figure 2). Although the FISH and qPCR results showed that XPi2 in some stages of the cell cycle might play different roles correlated with the expression of *XIST* in X chromosome inactivation, the different biological roles that small RNA plays from X chromosome inactivation need further investigation.

Ito *et al*. first proposed that gene expression could be functionally regulated by the direct correlation between small RNAs and mRNAs in human cells [44]. Ito's finding, which involves a small exogenous RNA, differs from our results. We found that a de novo endogenous small piRNA, located on the *XIST* genome without correlation with XCI (Supplementary Table S1 and Figure S1-3), coordinated with G4 DNA to influence structure formation. G4 DNA regions are viewed as therapeutic targets in oncology because they have the potential to activate or deactivate gene expression [45, 46]. Our bioinformatics analysis suggests that G-quadruplex motifs are prevalent throughout the genome (Supplementary Figure S1-1 and S1-2). Therefore, there is an increasing demand to understand their functions. Human oncogenes, including transcription factors, increase cell growth, proliferation, differentiation, and apoptosis, and it is likely that the up-regulated oncogenes in cancer cells are caused by over-expression or deregulation of miRNAs [47–49].

Many promoters of oncogenic genes associated with G-quadruplexes have emerged as therapeutic targets. The most interesting part is that G-quadruplexes need to work with small RNA molecules to stabilize these structures. For example, the *Myc* proto-oncogene is overexpressed in up to 80% of solid tumors controlled by a microRNA molecule [45]. Therefore, how to detect the changes of the G4 structure with XPi2 is a very critical issue in our study. Although CD and NMR are very traditional ways to detect G4 structures, the CD method still is a convincing method to detect the specific changes in guanines of the parallel-stranded G-quadruplex via various peaks which switched to the antiparallel-stranded. When the G4 structure was combined with the small molecule, it was still detectable [50]. The NMR spectra of oligonucleotides can usually record the H and 2H exchange conditions via measuring the changes of the G-rich DNA sequences forming four-stranded structure G-quadruplexes from antiparallel to parallel.

Lu and Tsourkas detected miRNAs by using locked nucleic acid-enzyme-labeled fluorescence - fluorescence in situ hybridization (LNA-ELF-FISH) and found that miRNAs were primarily located in the cell cytoplasm regardless of the cell cycle [51]. However, our results indicated that XPi2 appeared in the nucleus during certain stages of the cell cycle but not the metaphase. LNA-ELF-FISH is a highly sensitive and specific method for short sequence detection. Therefore, an individual piRNA is identified as a bright photostable fluorescent spot when LNA-ELF-FISH is applied. This method is better than other kinds of FISH methods (Figure 2).

It has been reported that hnRNP A1 destabilizes both the human telomere and the *KRAS* G-quadruplex (Please refer to H Fukuda, 2002 and M Paramasivam, 2009), and that nucleolin facilitates G-quadruplex formation [17, 52], whereas hnRNP A1 does not. According to Ito's experiment model, which is applied in this study, we found the expression of both hnRNP A1 and nucleolin changed after the disassembly of the DNA G-quadruplex structure (Supplementary Figure S2-2) [53]. The model was proposed (Figure 6B); however, at present, there might not be forceful data that support our proposed model; nonetheless, separate experiments demonstrate that XPi2 alters *KRAS* expression via proteins by regulating the structure of *KRAS* G-quadruplex (Figures 4E, 4F: The expression of the *KRAS* changed after sh*hnRNPA1* and sh*nucleolin* were treated.). The interaction between XPi2 and *KRAS* DNA G-quadruplex formations has been confirmed from the results of CD and NMR (Supplementary Figures S3 and Figure 5D).

The stability of G-quadruplex structures is determined by certain proteins [54]; for instance, G-quadruplex structures are connected by alternatively spliced pre-mRNA sequences with their binding proteins, including BRCA1, hnRNP A1, hnRNP D, and POT1 [55]. In most somatic cells, telomeres shorten when cells divide, and hnRNP A1 might contribute to the telomerase function by its ability to unwind the G-quadruplex structures of telomeres [54]. In this study, we found that a small non-coding RNA, XPi2, altered the formation of *KRAS* DNA G-quadruplex and influenced cancer cell behavior (Supplementary Figures S4A and S4B). XPi2 recruited the proteins nucleolin and hnRNP A1 to pinpoint the *KRAS* promoter (Figure 4E and 4F) and interrupted *KRAS* DNA G-quadruplex formation.

In comparison with DNA quadruplexes, RNA quadruplexes are preferentially formed in parallel conformation due to anti-geometry of the glycosidic bond in ribonucleosides. In contrast, the DNA G-quadruplexes adopt both parallel and antiparallel structures that can often switch from one to the other, depending on experimental and sequence conditions. Besides, the DNA G-quadruplex folding may be influenced by salt concentration [55]. Although both RNA and DNA quadruplex structures may provide important insight into cell processes and disease development, the existence of RNA G-quadruplexes *in vivo* is rarely correlated with promoter regions of oncogenes compared with DNA quadruplexes [55].

Recent studies regarding G-quadruplexes have demonstrated their biological roles in the guanine-rich strands of *c-myc, c-kit* and variant *bcl-2* oncogene promoters [45]. The significance of G-quadruplex in gene regulation has been observed in various human diseases, including genetic diseases and cancers. Aberrant G-quadruplex formation in disease-associated genes demonstrates a new concept of gene regulation [25, 56]. *KRAS*, an oncogene, is one of the most commonly mutated genes in many cancers [57]. A role of mutated *KRAS* gene plays in therapeutic resistance merits significant attention [57, 58]. The interdependence between XPi2 and *KRAS* DNA G-quadruplex formations may have therapeutic implications. By the result of the expression of *KRAS* and XPi2 in 31 colon cancer cases, it was revealed that XPi2 potentially interacted with the *KRAS* gene in the 31 colon and nearby tissues. Decreasing XPi2 expression levels may stabilize G-quadruplex structures of the *KRAS* promoter. This result may down-regulate *KRAS* expression and reduce *KRAS* proliferated cancer cells despite *KRAS* oncogene mutations (Figures 5A and 6A; Supplementary Figure S4). G-quadruplexes are playing regulatory roles in gene promoter regions in many oncogenes and tumor suppressor genes, such as *C-MYC*, *C-MYB*, *HRAS, BCL-2, VEGF, PDGF, RB, and TERT* [11, 59–62]. G-quadruplexes are considered to be emerging therapeutic targets in cancer treatment because their structures can be harnessed to control transcription. Transcriptional repression of oncogenes occurs through G-quadruplex stabilization, whereas the transcription of tumor suppressor genes occurs via the disruption of G-quadruplex structures [45, 60, 63, 64]. Therefore, according to previous literature, the regulation of piRNAs may be one of the critical issues in tumorigenesis [65–68].

In this study, we have demonstrated that XPi2 alters *KRAS* expression by regulating the structure of *KRAS* G-quadruplex. When we transfected *hnRNP A1* or *nucleolin*, the XPi2 expression was changed in the cell line. In contrast, when we transfected the synthesized RNA, the migration and proliferation of cells would change. Accordingly, we suggest that some other piRNAs may also play similar roles in regulating gene expression. Our present work reveals that G-quadruplex-specific piRNAs may provide important insight into cancer therapy and lead to novel targeted therapies in treating cancer or other diseases, and pinpointing G-quadruplexes with piRNAs may be regarded as a novel anti-cancer strategy.

## MATERIALS AND METHODS

A particular small RNA with the sequence length of 31 nucleotides (nt) was obtained by the identified bioinformatics method [29, 69]. This 31 nt endogenous small RNA located on *XIST*, named XPi2, has three identical repeat sequences of XPi2 on the repeat A portion of *XIST* (Figure 1A) [70].

### Bioinformatics to identify *XIST*-derived endo-siRNAs and their target genes

Functional RNA Database (fRNAdb) hosts a large collection of known/predicted non-coding RNA sequences from various public databases. To predict the candidates of *XIST*-derived endo-siRNAs, we aligned the sequences of *XIST* with functional SiRNAs (fsRNAs), and the Illumina-Solexa experiments on human embryonic stem cells were conducted to verify these candidates. To identify the targets of *XIST*-derived endo-siRNAs, we modified our previous approach and verified them by two gene expression profiles (GDS596 and GSE5364) obtained from NCBI GEO. All of the sequences were collected from UCSC hg19 [71–73].

### Microarray hybridization

Total RNA was labeled according to the Gene Chip® Expression Analysis Manual as provided by the manufacturer (Affymetrix, Santa Clara, CA, USA) and hybridized to Affymetrix Human Genome U133 Plus 2.0 arrays (Affymetrix) for 16 h. After being washed and stained in Gene Chip® Fluidics Station 450, the intensities of the hybridized probes on the arrays were scanned using the Gene Chip® Scanner 3000 7G, and the data of individual cells were analyzed and computed using Gene Chip® Operating Software version 1.3 (Affymetrix) on a PC-compatible workstation.

### Discovery of the functions of XPi2 with gene expression profiles

To investigate the functions of XPi2, we mined the different expression profiles when XPi2 was knocked down, and when it was not. The expression values of the remaining probe sets were normalized and analyzed using the dChip2010 software (http://biosun1.harvard.edu/∼cli/dchip2010.exe) with PM/MM model7. We selected at least a 2-fold geometric change in gene expression profiles between the samples above. The functions of these genes were further examined by performing GO and KEGG pathway enrichment using the DAVID gene annotation scheme [74, 75].

### RNA extraction and real-time PCR

For each 24-well (2×10^5^ cells/well), total RNA was extracted from cultured cells and tissues. Total RNA was first isolated from cells according to the manufacturer's instructions of Trizol (Invitrogen, Carlsbad, CA, USA) and the standard protocol. To detect the concentration of total mRNA, a Nanodrop spectrophotometer (Gene) was utilized. Reverse transcription (RT) was conducted with the High-Capacity cDNA Reverse Transcription Kit (Thermo Fisher Scientific). Roche LightCycler 480 (Roche) was used to achieve the amplification reaction, and the protocol was carried out at 95°C for 10 min and 50 cycles at 95°C for ten secs, 60°C for 30 secs and 72°C for 10 sec. The relative expression was normalized to the expression of *GAPDH* RNA. All RT-PCR reactions were performed in triplicate.

### Real-time quantitative-PCR and RNA expression

RNA isolation from specimens or cultured cells and reverse transcription were performed as described. The reverse-transcriptase quantitative polymerase chain reaction (RT–qPCR) analysis of XPi2 in normal cells, Huh-7 cells, and XPi2-expressing Huh-7 stable cell lines were performed using SYBR Green with the ABI 7500 Real-Time PCR System (Applied Biosystems). RT–qPCR of XPi2 (31 nt) and the *KRAS* gene in paired colon tumor and non-tumor tissues were performed using a LightCycle 480 (Roche, Mannheim, Germany) with a primer/probe system. The sequences of XPi2 and the various genes were evaluated to design specific forward and reverse primers shown in Supplementary Table S3.

RT–PCR of XPi2 in Huh-7 stable cell lines was performed using a TaqMan MicroRNA Assay designed according to the manufacturer's instructions (Applied Biosystems) following isolation of small RNA with the mirVana miRNA Isolation Kit. *U6* small nuclear RNA was used as an internal control. All RNA expression levels were normalized to *GAPDH* (glyceraldehyde-3-phosphate dehydrogenase) RNA with the Ct method according to Liu et al. [76, 77].

### RNA and LNA fluorescence in situ hybridization (FISH) and immunofluorescence

Interphase and metaphase spreads were prepared for FISH using standard methods. DNA FISH probes (antisense of XPi2: GACCTTATTCAGATGGGATGGGCCATGTGCA; antisense of a scramble: CTGGAATAAGTCTACCCTACCCGGTACACG) were labeled with fluorescein or Cy5 dyes using the Label IT Labeling Kit (Mirus, Madison, WI, USA).

XPi2 RNA/LNA Antisense-RNA: UGCACAUGGCCCAUCCCAUCUGAAUAAGGUC) and *XIST* RNA (Antisense RNA: rArArG rGrArU rGrGrG rArArC rArArA rGrCrA rArGrG rCrCrU rGrGrA rCrCrU rG) /DNA ((Xq13.2) from Cytocell Ltd.) FISH probes of the commercially synthesized products were also used (Supplementary Table S3). LNA and DNA FISH: The water bath was preheated to 73°C and ready to use, and then a solution of the one μl paint probe, seven μl hybridization buffers, and two μl H2O was denatured at 72°C for five minutes. The slides were denatured in a buffer solution (70% formamide, 2x saline sodium citrate (SSC)) at 72°C for two minutes and then quenched in ice-cold 70% ethanol for five minutes. This procedure was repeated three times before hybridization. RNA FISH: The slides after fixation by 1% formaldehyde were inserted into solution A (80% formamide, 10% 1X saline sodium citrate (SSC), and 10% H2O) at room temperature for two minutes. Then they were quenched in ice-cold 70% ethanol for five minutes to fix the slides. Standard methods were used for both kinds of probes. The slides were dehydrated, air-dried and preheated to 37°C for two minutes before probe addition. The slides were incubated overnight at 37°C in a humid chamber in an incubator. After hybridization, the slides were removed and washed three times in a 50% formamide, 2x SCC buffer for five minutes at 72°C (LNA or DNA FISHs only) or room temperature, respectively. Then, the slides were washed twice in a PN buffer at room temperature, and eight μl DAPI (4, 6-diamidino-2-phenylindole) (1 mg/ml, Abbott, IL, USA) was added to the slides, and at least 100 interphases, as well as metaphases, were studied in each slide. Each case was performed on a NikonE600 microscope with the cytovision software [78].

### Cell proliferation, wound healing and invasion assays

We investigated the effects of XPi2 on cell viability, invasion, and migration. After silencing XPi2 for 72 h, we studied the viability of Huh-7 cells with an MTT assay according to the previous study by Yeh et al. [79]. Cell mobility was measured using wound healing and transwell invasion assays, respectively [79]. 5 × 10^5^ cells with silenced XPi2 were seeded in a 12-well plate to conduct a wound healing assay. Briefly, a 200 μl pipette tip was used to create a 1-mm gap area in confluent culture of cells which were incubated for 24 h and 48 h, respecitvely Afterwards, the gaps were photographed using an inverted phase-contrast microscope (TE2000-U; Nikon, Tokyo, Japan), and the cellular migration was analyzed using the software “TScratch.”. For assessing the cellular invasion, 5 × 10^5^ cells with silenced XPi2 were cultured in a serum-free medium and then added to the upper compartment of a Boyden chamber slide (8-μm pores, Greiner, Frickenhausen, Germany). 50% Fetal bovine serum was added as a chemoattractant in the lower chamber. After 24 h, non-invaded cells in the upper face of the filter were removed. The invaded cells in the lower face of the filter were fixed with 0.5% glutaraldehyde and stained with Giemsa. All cells for each well were counted.

### Cell culture and gene knockdown

Huh-7, MCF-7, and normal lymphoid cells were grown in either the Dulbecco's modified Eagle medium or Roswell Park Memorial Institute 1640 medium (DMEM or RPMI, Invitrogen, Carlsbad, CA, USA) supplemented with 20% fetal bovine serum (FBS, Biological Industries, Great Island, NY, USA), 100 U/mL penicillin, and 0.1 mg/mL streptomycin (Invitrogen, Carlsbad, CA, USA). The cell harvesting procedures were performed with 0.1 μg/mL colcemid and 65 ng/mL nocodazole (Sigma, Chemical Co., MI, USA). Short hairpin RNAs (shRNAs) pinpointing human *DICER1*, *AGO3*, *nucleolin*, or *hnRNP A1* were transfected into cells using LipofetamineTM 2000 (Invitrogen, Carlsbad, CA, USA) according to the manufacturer's protocol. PLKO.1-shLuc (shLuc) was used as the control shRNA. It was transfected in 16, 24, and 48 h. For XPi2 knockdown, antisense oligonucleotide sequences of XPi2 (100μM; 5’ - GACCTTATTCAGAT GGGATGGGCCATGTGCA) or scrambled control sequences (100μM; 5’ - CTGGAATAAGTCTACCCT ACCCGGTACACG) were transfected into cells. The gene knockdown was confirmed with RT-qPCR and RNA-FISH.

### Cell transfection

The shRNA plasmids were transfected with LipofetamineTM 3000 (Invitrogen). For each 6-well (8×105 cells/well), 6.4 μg plasmids were diluted in 200 μl Opti-MEM followed by adding six μl P3000. Meanwhile, the six μl LipofetamineTM 3000 was also diluted in the 200μl Opti-MEM. The diluted LipofetamineTM 3000 was added to the diluted plasmids and incubated for 5 min at room temperature. The mixture was added dropwise to the cell cultures and mixed gently. The cells continued to be incubated for 18 or 24 h for further downstream analysis.

### Protein extraction and western blotting

A modified RIPA buffer (50 mM Tris–HCl, 150 mM NaCl, 0.25 % SDS, 1 % Triton X-100, 0.25 % sodium deoxycholate, 1 mM EDTA, 1 mM EGTA, 1 mM dithiothreitol) with protease inhibitor cocktail (Sigma) was used for protein isolation. Protein concentrations were determined using the Bio-Rad Protein Assay kit (Bio-Rad). Cell lysates containing 30 μg of total protein were resolved in 8 % polyacrylamide gels and transferred to Immun-Blot® PVDF membranes (Bio-Rad) and blocked with 5 % albumin, bovine (AMRESCO) in PBST (0.1 % Tween 20 in PBS). The membranes were probed at 4°C overnight with primary antibodies against hnRNP A1 (1:1000 dilution, Sigma), nucleolin (1:1000 dilution, Abcam), and beta-actin (1:5000 dilutions, Abcam). The membranes were subsequently washed three times with PBST to remove excess primary antibodies, and incubated with appropriate HRP-conjugated secondary antibodies (1:5000 dilution). All antibodies were purchased from Clarity™ Western ECL Substrate (Bio-Rad). Signals were visualized by the ChemiDoc-It (UVP).

Western blot antibody:

Anti-hnRNP-A1 antibody (R4528) | SIGMA, Mouse monoclonal http://www.sigmaaldrich.com/catalog/product/sigma/r4528?lang=en&region=TW

Anti-Nucleolin antibody [EPR7951] (ab134164) | Abcam http://www.abcam.com/nucleolin-antibody-epr7951-ab134164.html

Anti-beta Actin antibody [mAbcam 8226] (ab8226) | Abcam http://www.abcam.com/beta-actin-antibody-mabcam-8226-ab8226.html

sh*hnRNPA1* TRCN0000006586

sh*NCL(nucleolin)* TRCN0000062283

sh *AGO3* TRCN000007869

sh*DICER1* TRCN0000051260

http://rnai.genmed.sinica.edu.tw/searchDatabase.

### Northern blots

Total RNA was extracted from cell lines with the NE-PER Nuclear and Cytoplasmic Extraction Kit (Thermo). 50 ug of total RNA were dissolved in a gel loading buffer (10 mM mM EDTA ph 8.0, 96% (v/v) formamide, 0.01% xylene cyanol and 0.01% bromophenol blue), heated at 95°C for 5 min, loaded onto denaturing 12% urea-PAGE, and separated for 2 h at 150V and then transferred onto the nitrocellulose membrane (Pall Corporation, East Hills, NY, USA) followed by a crosslinking with UV irradiation. The RNA blots were prehybridized at 42°C for 3h using DIG Easy Hyb Granules (Roche) and subjected to hybridization with the DIG-LNA probe

(LNA probe: /5DIGN/TTATTCAGATGGGATGGGCCATGT/3DIG_N/24 bp) for XPi2 (10 pmol) at 42°C overnight. Following hybridization, the membranes were rinsed and then washed sequentially with 2X SSC/0.1% sodium dodecyl sulfate (SDS), 0.1X SSC/0.1% SDS at 42°C. Detection was performed using the DIG Northern Starter Kit (Roche) according to the manufacturer's instructions. In brief, the membranes were blocked in a blocking buffer for 30 min and then incubated with an alkaline phosphatase-conjugated anti-DIG antibody for 30 min followed by washing three times with a washing buffer. After equilibration in the detection buffer, the blots were incubated with chemiluminescent substrate CDP-Star and visualized by the ChemiDoc-It (UVP).

### RNA-CHIP and CHIP-seq

ChIP assays are performed according to the Active Motif protocol. In brief, cells are grown to 90% confluence in 150-mm dishes and lysed in an ice-cold complete lysis buffer on ice for 30 min after crosslinking. Pelleted nuclei are suspended in a complete shearing buffer. Chromatin is sheared into 100 bp to 1000 bp fragments by sonication. Chromatin is treated with a reagent (DNase I) for a more efficient RNA-CHIP. Ten micrograms of total chromatin are incubated overnight at 4°C with the antisense of the XPi2 probe (GACCTTATTCAGATGGGATGGGCCATGTGCA). After washing by a buffer, the immune complex is eluted by an elution buffer, and two μl of 5 M NaCl is added to reverse the formaldehyde cross-linking at 65°C for 1.5 h. Following incubation with proteinase K, RNA is recovered by phenol/chloroform and ethanol precipitated. RNA extracts are treated with a reagent (DNase I) to eliminate genomic DNA contamination and analyzed by real-time PCR. According to the manufacturer's recommendations, the PCR is performed in a final volume of 20μl using a LightCycler instrument (Roche Diagnostics).

### Circular dichroism (CD)

CD spectra were obtained using a JASCO J-600 spectropolarimeter equipped with a thermostatted cell holder. The samples containing 50 μM RNA and DNA solutions for each, XPi2, *KRAS*, and XPi2-*KRAS* were used in the CD experiments in 50 mM Tris, pH 7.2 and 100 mM KCl. Spectra were recorded in a 5-cm quartz cuvette. A thermometer was inserted into the cuvette holder to allow a precise measurement of the sample temperature. The spectra were calculated with the J-700 Standard Analysis Software (Japan Spectroscopic Co., Ltd) and were shown with ellipticity expressed in mill degrees (mdeg). Each spectrum was recorded three times, smoothed and subtracted to the baseline [28].

### NMR experiments

The oligonucleotides of *KRAS* and XPi2, at least 5 μM, were annealed in a buffer containing 150 mM KCl, 25 mM KH_2_PO_4_, 1 mM EDTA, pH 7.0 at 95°C for 10 min followed by slow cooling to room temperature. The experiments were performed on a Bruker AV600 spectrometer (equipped with a cryoprobe). Typical acquisition conditions for an NMR spectrum were 90° pulse length, 2.0 s relaxation delay, 32 K data points, 16 ppm spectrum width, 64–128 transients, and each sample in 90% H_2_O/10% D_2_O with 150 mM KCl, 25 mM KH_2_PO_4_, one mM EDTA, pH 7.0. Each solution was titrated directly to the DNA solution inside an NMR tube [80]. The spectra were recorded utilizing a standard jump-return pulse sequence (298 K) for water suppression with a relaxation delay of 2.0 s [81].

### Statistical analysis

Student's t-test was used for analysis of assays. Significance was accepted at P<0.05.

### Case collection

Resected primary colon cancer and nearby non-cancerous tissue samples (n=62) were obtained from 31 patients at the Kaohsiung Medical University Hospital. The tumor tissues were frozen immediately after surgical resection and then stored in liquid nitrogen until extraction of either RNA or DNA. The consent of the patients was obtained, and the Institutional Review Board of Kaohsiung Medical University Hospital approved the protocol.

## SUPPLEMENTARY MATERIALS FIGURES AND TABLES








