# Supplementary material for: An XIST-related small RNA regulates KRAS G-quadruplex formation beyond X-inactivation
Source: Oncotarget. 2016 Nov 17;7(52):86713–29. doi: 10.18632/oncotarget.13433 (PMC5349948; doi:10.18632/oncotarget.13433)
Supplement: Supplementary file 1 [file oncotarget-07-86713-s001.pdf]

An *XIST*-related small RNA regulates *KRAS* G-quadruplex formation beyond X-inactivation

SUPPLEMENTARY FIGURES AND TABLES

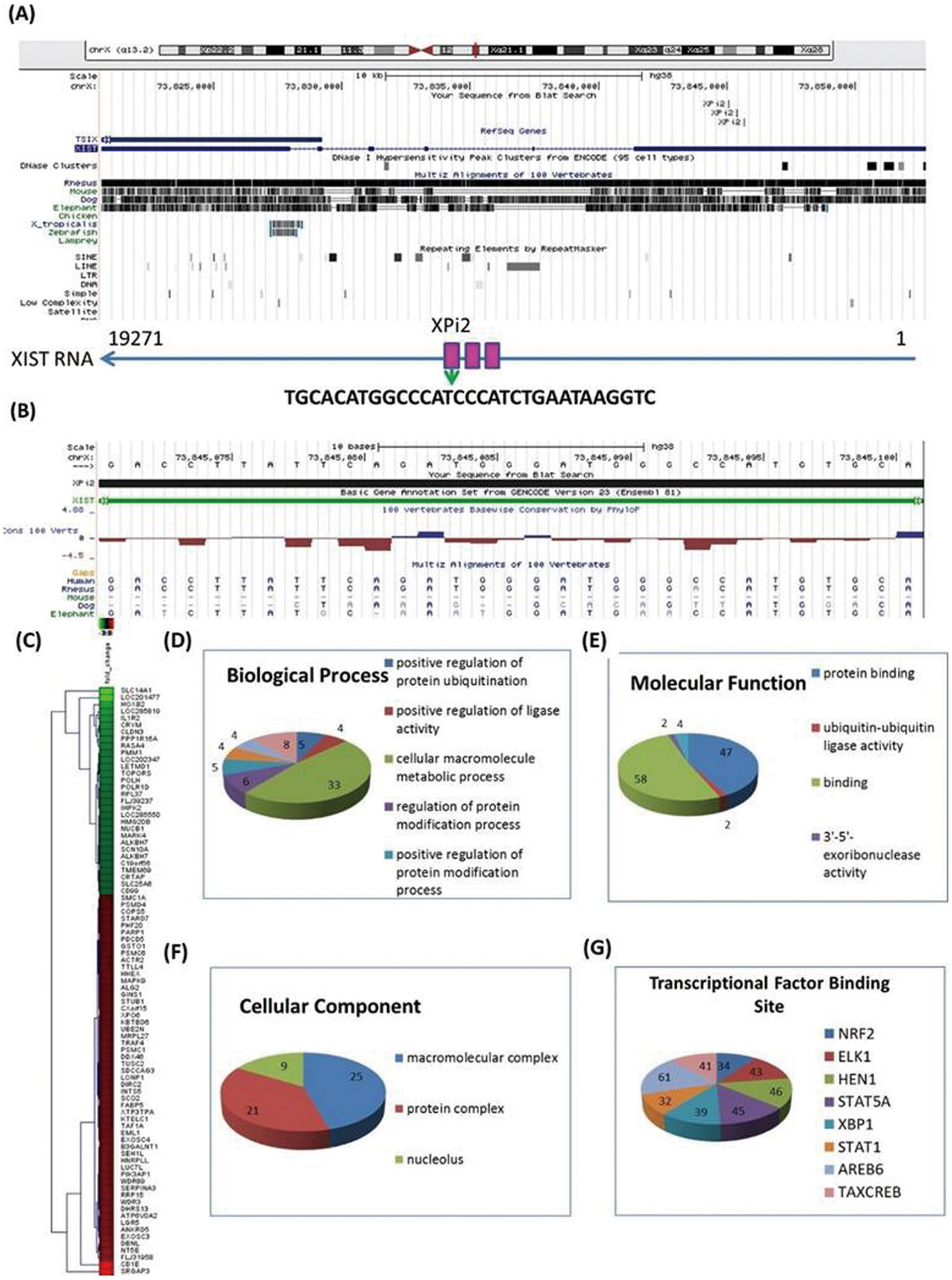

Supplementary Figure S1-1: The genomic view of *XIST* and XPi2. A. *XIST* RNA was located on chromosome X and conservation of cross-species. **B.** *XIST*-derived XPi2 (pink box) with the length of 31bp was highly conserved in vertebrates. **C.** The Gene expression profile and functional analysis of knockout XPi2: Hierarchical clustering analysis was presented as fold change (more than 2-fold changed) of knockout XPi2 versus control expression levels. **D.** Gene Ontology-Biological Process **E.** Gene Ontology-Molecular Function **F.** Gene Ontology-Cellular Component **G.** Transcriptional factor binding site (p-value<0.001)

Term	Count	%	P-Value	Genes
Telomere Maintenance	4	7.84	4.69E-04	HIST1H2AC,HIST1H2BJ,H2AFX,HIST1H3D,HIST1H3H
Sprouty regulation of tyrosine kinase signals	2	3.92	3.10E-02	HRAS,SPRY1

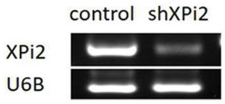

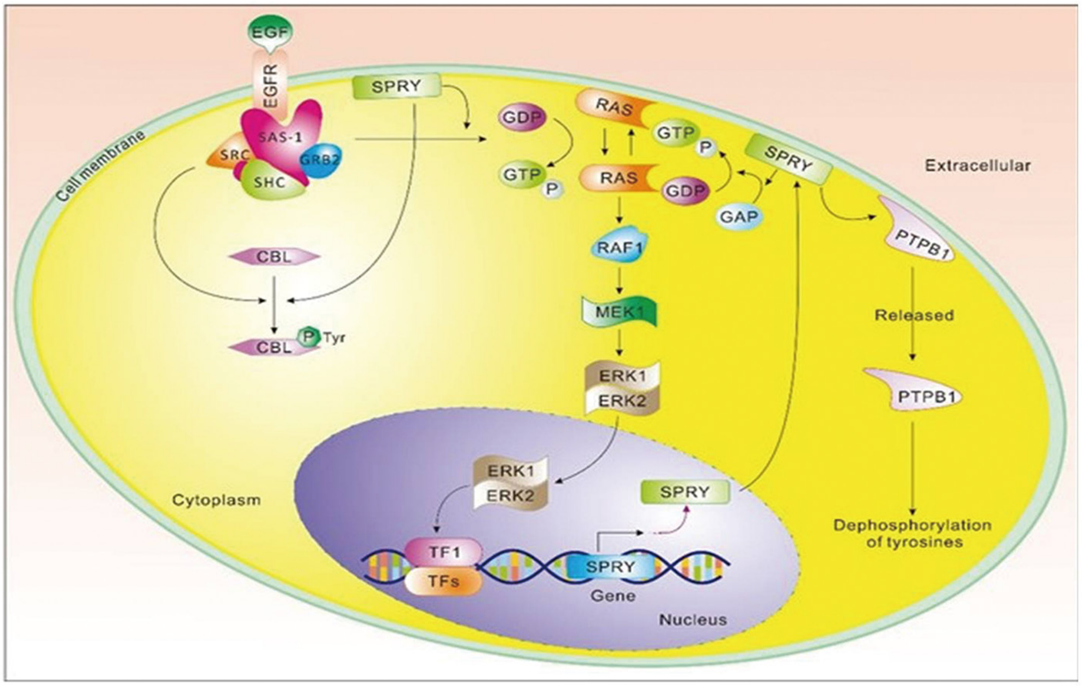

Supplementary Figure S1-2: The gene expression of XPi2 array data. The efficiencies of XPi2 analyzed by Gene-Express-Based determination according to the user's manual (Affymetrix Inc) were compared before and after XPi2 was knocked down. It showed the significance of the pathway of down–regulated genes after XPi2 was knocked down.

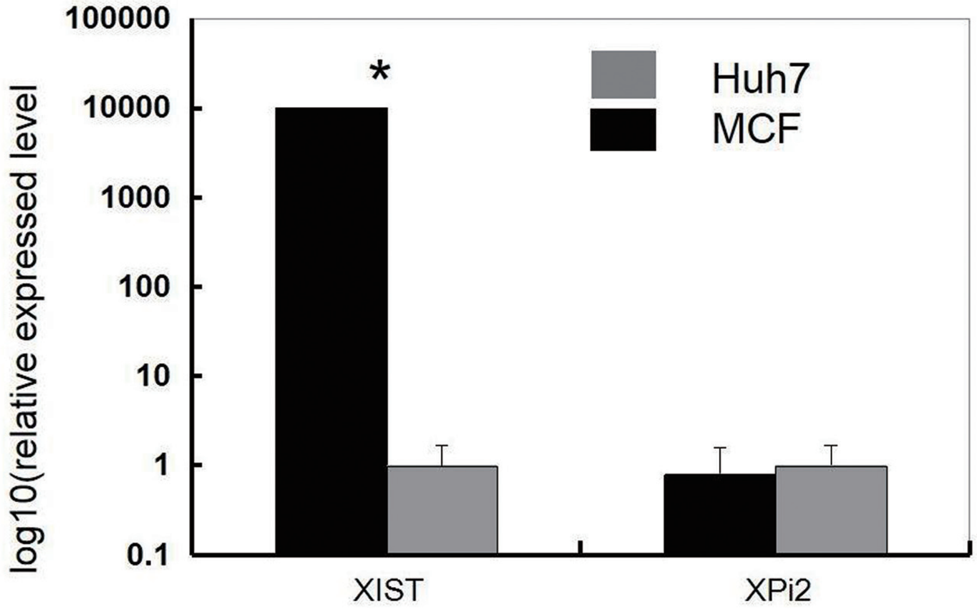

Supplementary Figure S1-3: Quantitative RT-qPCR was performed, and it was showed that the expression level of XPi2 was not correlated with the expression of *XIST* in the tested cell lines. (*P < 0.05 for the control versus *XIST* and XPi2 in two different cell lines)

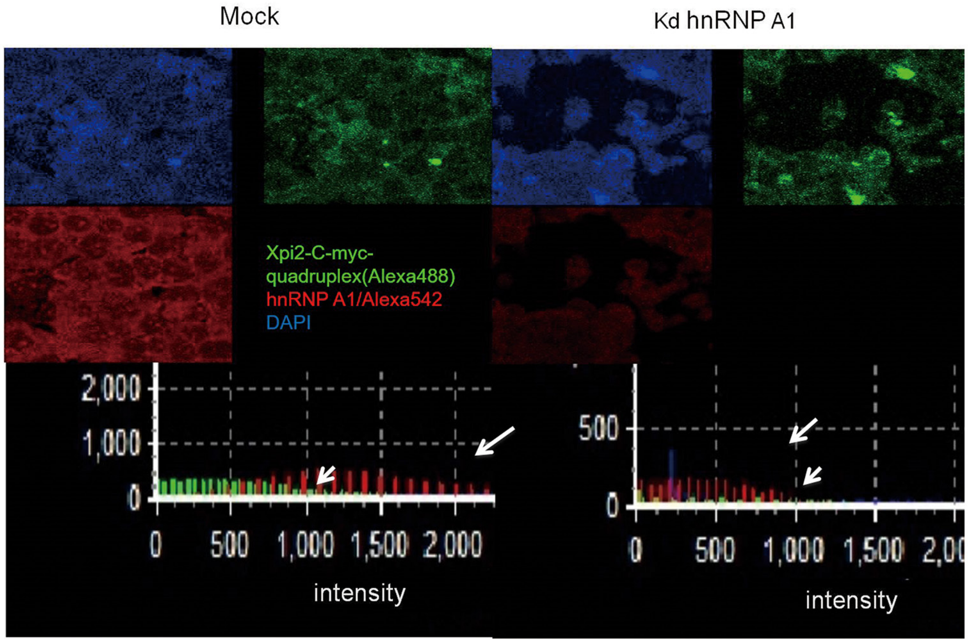

Supplementary Figure S2-1: After protein (hnRNP A1) was knocked down (fluorescent intensity from 2000 to 1000), XPi2 could not disrupt the formation of *C-MYC* G-quadruplex, and the density (1000) *C-MYC* G-quadruplex was showed; the result of the mock showed the same density (1000) *C-MYC* G-quadruplex (Kd= knockdown). The result showed that there was no interaction between XPi2, hnRNP A1, and *C-MYC* G-quadruplex.

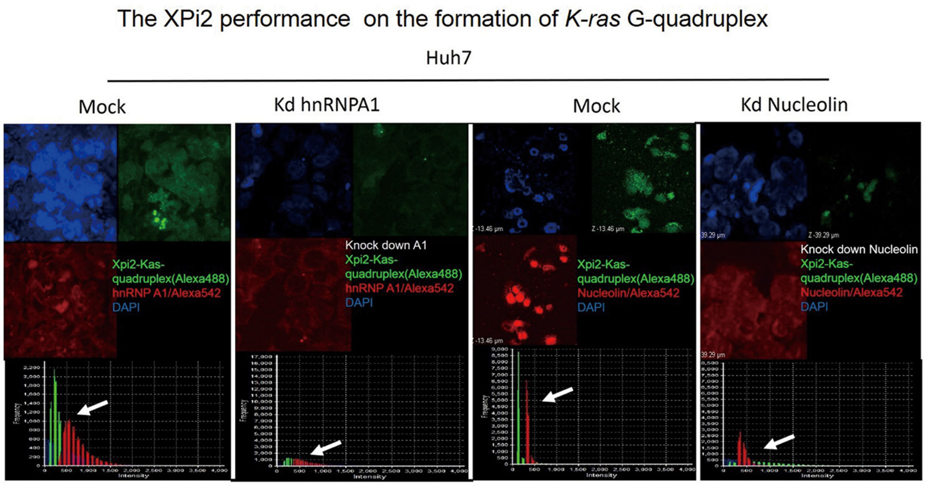

Supplementary Figure S2-2: After both proteins had been knocked down, XPi2 disrupted the formation of *KRAS* G-quadruplex and showed the presence of the low-density *KRAS* G-quadruplex; on the contrary, the result of the mock showed the presence of the high - density *KRAS* G-quadruplex (Kd=knockdown).

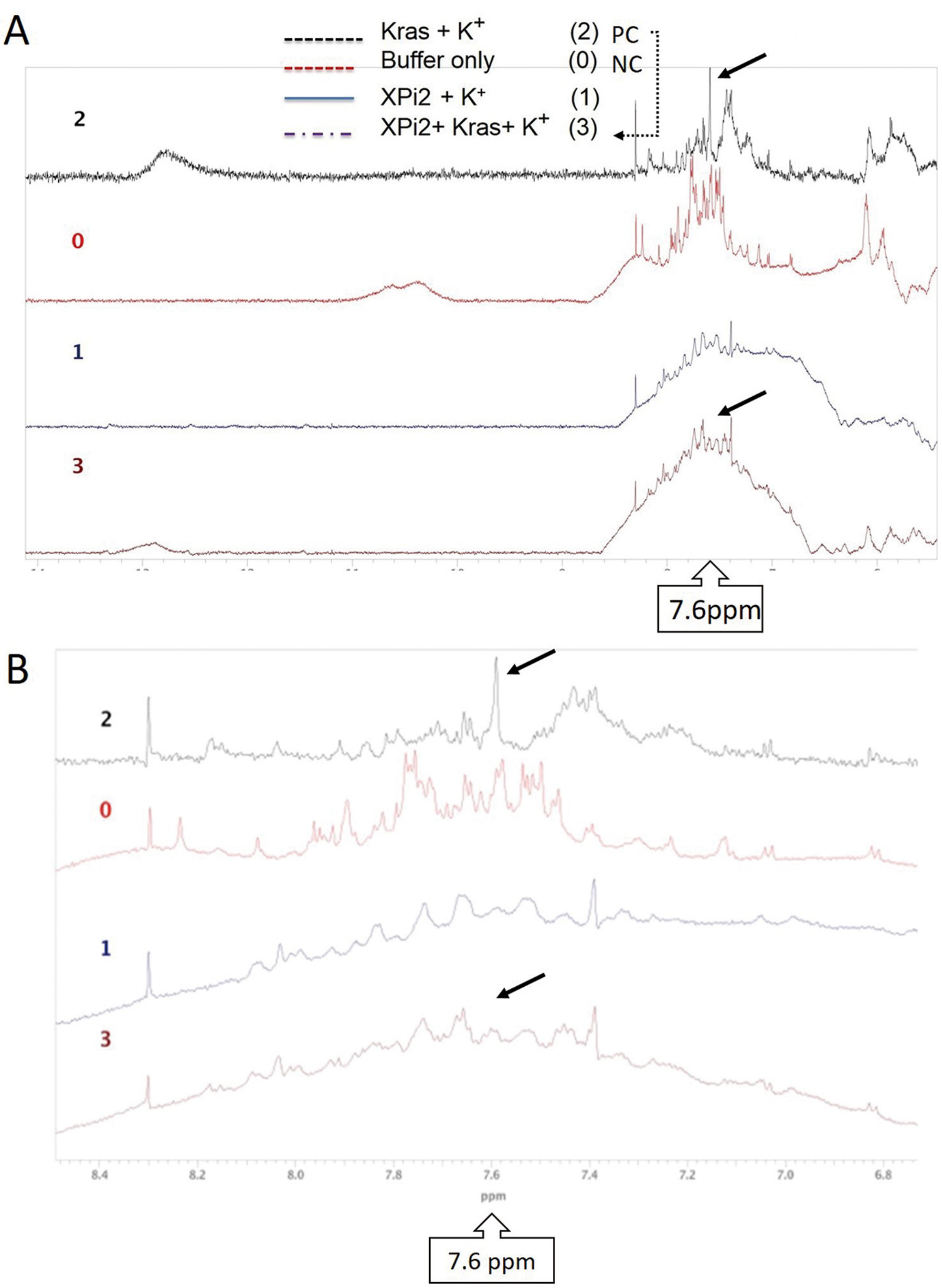

Supplementary Figure S3: A. The NMR experiment showed three different conditions (1, 2, 3) and one for the negative control (0).1=XPi2, 2=*KRAS*, 3=XPi2 + *KRAS*, the 1, 2, and 3 are in equilibrium between their free and bound states. **B.** Enlarged (A) peaks in 7.6 ppm: The NMR spectra of oligonucleotides are usually recorded at room temperature under the H and 2H exchange conditions to measure the change of the G-rich DNA sequences forming four-stranded structure G-quadruplexes from antiparallel to parallel. Rapid motions for the NMR spectra of oligonucleotides with K+ are evident by the positive peaks, which are contoured 2, and resonance peaks are observed in the 7.6 ppm region in the hydrogen dimension. However, the negative signals are contoured 3, which monitored the existence of XPi2 reaction, and they were the same negative peaks only when the buffer conditions (contoured 0) were present.

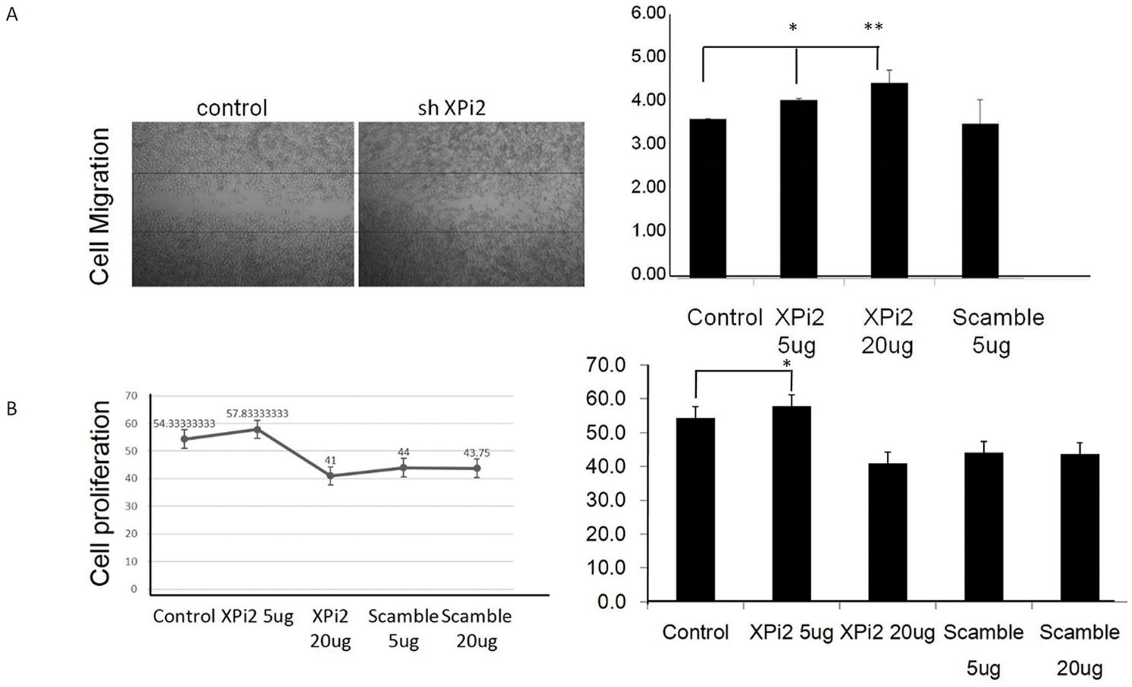

Supplementary Figure S4: A. XPi2 played a crucial role in cell biology to change cell migrations. The transwell invasion assay results were shown. The different results of the wound-healing assay in the Huh-7 cell line between the control and XPi2 after the silence down for 72 h were shown on the right and left panels. **B.** After the knockdown of XPi2, cell proliferation was slightly increased in 5ug and decreased in 20ug (*P < 0.05,* *P<0.001 for the control versus XPi2-knockdown treatment in the Huh7 cell line respectively).

Supplementary Table S1: Quantitative RT-qPCR was performed, and the results were shown in the upper table. It was showed that the expression levels of *XIST, TSIX, OCT, NANOG, SOX2, MYC, KLF4,* and *GAPDH* were measured in various cell lines (The labels on the top were aligned accordingly as follows: The A549, H1299, Huh7, HepG2, SW620, HT29, HS578, MCF2, K562, HL60, GBM, and TE671, respectively.). (The result of the cycle No. showed different expression levels among different cell lines beyond gender; the control versus each gene in different cell lines) Quantitative RT-qPCR was performed (*XIST, TSIX* and *GADPH*; XPi2 and *U6B*), and the results were shown in the lower table, and it was showed that the expression level of XPi2 was not correlated with the expression of *XIST* in two different cell lines. (The control versus *XIST* and *TSIX* in different gender cell lines). The statistical information and results were shown in Figure S1-3

See Supplementary File 1

Supplementary Table S2: The Nucleolin and hnRNP A1 were specifically bound to XPi2 RNA after the protein pull-down assay via the performed proteomic analysis compared with the human control RNA

See Supplementary File 2

Supplementary Table S3-1: Analytical sequences of the relative XPi2 and XPi2 within the primers

See Supplementary File 3

Supplementary Table S3-2: RNAi cord knock down sequences

TRC/RNAi Core knockdown								
Symbol	Clone ID	Clone Name	Vector	Sequence	Species	Knockdown Efficiency	Cell	Flag*
hnRNPA1	TRCN0000006586	NM_002136.1-421s1c1	pLKO.1	AGATATTTGTTGGTGGCATTA	human	84%	A549	Good
NCL	TRCN0000062283	NM_005381.2-2396s1c1	pLKO.1	CCTTGGAAATCCGTCTAGTTA	human	88%	A549	Good
shCon	TRCN0000208001	pLKO.1-emptyT	pLKO.1	CCGGACACTCGAGCACTTTTTG	human	n/a	n/a	n/a

Supplementary Table S3-3: QPCR primer sequences

QPCR primer:				
**Transcript**	**Name**		**Product size**	**Probe #**
NM_002136.2	hnRNPA1	F GAAGTGATTGAAATCATGACTGACC	98	59
		R ATCTTATCCACGGAGTCATGGTCG		
NM_005381	Ncleolin	F AGAAGGAAATGGCCAAACAG	78	17
		R AAGCCGTAGTCGGTTCTGTG		
NM_033360.3	KRAS	F TGGACGAATATGATCCAACAAT	121	62
		R TCCCTCATTGCACTGTACTCC		
NM_002046.5	GAPDH	F AGCCACATCGCTCAGACAC	66	60
		R GCCCAATACGACCAAATCC		
